# RISK FACTORS ASSOCIATED WITH FRAILTY STATUS CHANGE: A PROSPECTIVE STUDY IN A GERIATRIC OUTPATIENT SETTING

**DOI:** 10.1093/geroni/igae098.2771

**Published:** 2024-12-31

**Authors:** Chiung-Jung Wen, Chung-Sheng Lee, Wei-Jia Huang, Ching-Huang Lin, Ding-Cheng (Derrick) Chan

**Affiliations:** National Taiwan University Hospital, Taipei, Taipei, Taiwan (Republic of China); Kainan University, Taoyuan, Taoyuan, Taiwan (Republic of China); National Taiwan University Hospital, Taipei, Taipei, Taiwan (Republic of China); National Taiwan University Hospital, Taipei, Taipei, Taiwan (Republic of China); National Taiwan University Hospital, Taipei, Taipei, Taiwan (Republic of China)

## Abstract

**Background:**

There has been limited research on the factors that predispose individuals to changes in frailty status. This study aims to identify the risk factors that lead to changes in frailty status among older adults.

**Methods:**

Participants were recruited from a prospective geriatric outpatient cohort at a medical center. Frailty status was evaluated at baseline and during a 1-year follow-up using a modified version of Fried’s criteria. Additionally, demographic characteristics, health status, and geriatric assessments were collected.

**Results:**

The mean age of the 140 participants was 79.5 ± 7.3 years. During the one-year follow-up period, 47.1% of participants experienced a worsening of frailty status, while 12.9% showed improvement, and 40% remained at the same frailty status. After adjusting for confounding variables, individuals who experienced more than one fall in the past year tended to exhibit deteriorating frailty status (□ □= -0.226, p = 0.005). The absence of teeth, which affects eating habits, also correlated with deteriorating frailty status (□ = -0.144, p = 0.061). Conversely, individuals with higher scores on the Mini-Mental State Examination tended to show improvements in frailty status □ = 0.198, p = 0.027).

**Conclusion:**

Our findings could contribute to the development of interventions and strategies designed to prevent and manage frailty in older adults.

